# Keeping it credible in cohort multiple Randomised Controlled Trials: the Community Ageing Research 75+ (CARE 75+) study model of patient and public involvement and engagement

**DOI:** 10.1186/s40900-016-0044-9

**Published:** 2016-08-30

**Authors:** Anne Heaven, Lesley Brown, Marilyn Foster, Andrew Clegg

**Affiliations:** Academic Unit of Elderly Care and Rehabilitation, Bradford Institute for Health Research, Bradford Royal Infirmary, Duckworth Lane, Bradford, BD9 6RJ UK

**Keywords:** cmRCT, Public, Involvement, Engagement, Participation

## Abstract

**Plain English summary:**

There are well documented benefits to involving patients and the public in research. However, there is little research published about their involvement in large complex studies such as cohort multiple Randomised Controlled Trials (cmRCTs). The cmRCT method establishes a group of participants, with a common characteristic (e.g. older people) who will be followed over a number of years. Other (sub) studies can also recruit from this pool of people. This method offers researchers many advantages, including being able to recruit from more hard to reach groups. However, cmRCTs also have features which can make it more complicated to involve patients and the public. For example more than one study may take place at the same time; studies may be spread out over a large geographical area and they may include a wide range of topics. In spite of these difficulties we have developed a way of working with patients, the public and researchers that provides stability over time but allows flexibility along the way. Our model of working has saved us time and money; helped us to recruit more widely, and enabled us to focus our research in areas that are important to older people with frailty.

**Abstract:**

**Background**

There is increasing guidance on how to make the most of the rich seam of data provided by large cohort studies, and growing recognition of the benefits of cohort multiple Randomised Controlled Trials (cmRCT) in health research. In contrast, there is a lack of discussion about patient and public involvement and engagement (PPIE) in these large and complex research infrastructures. Our aim was to create a structure to enable meaningful, sustainable public involvement within the cmRCT framework. We have established a core reference group of four key individuals with extensive links to other relevant local community structures and individuals.

**Results**

Using the CARE 75+ model we have engaged with a wide variety of patients and the public in a relatively short space of time. Activities have included scrutiny of protocols and assessment tools, and process evaluations; resulting in system efficiencies, increased recruitment and a more focused research agenda.

**Conclusions**

There is a need for strong public oversight and flexible models of PPIE in cmRCTs. The model of PPIE developed in the Community Ageing Research 75+ study presents one potential way to foster expertise and enable diversity.

## Background

This paper is based on the experience of running the Community Ageing Research 75+ (CARE 75+) study (ISRCTN16588124). This study is part of a programme of studies investigating the Primary Care Based Management of Frailty in Older People as part of the National Institute for Health Research Collaboration for Leadership in Applied Health Research and Care (NIHR CLAHRC) in Yorkshire and the Humber [1].

The central tenet of this paper is that new models of PPIE will need to be developed to ensure meaningful, sustainable, public oversight in cohort multiple randomised controlled trials (cmRCT). The discussion will draw on our own experience of developing the CARE 75+ study and in so doing we will briefly outline:

1. the CLAHRC Frailty theme
2. development of the cmRCT methodology, and
3. the unique proposition that cmRCTs present for public engagement.

Frailty is a condition characterised by loss of biological reserve and vulnerability to adverse outcomes [2]. The current health and social care response to frailty is largely reactive to acute crises and is predominantly located in secondary care. We have established the Primary care-based management of frailty theme as part of the NIHR CLAHRC programme [1]. The aim of this work is to enable a new approach to frailty that is proactive and preventative, predominantly located in primary and community care.

A key component of our NIHR CLAHRC programme of work has been the Community Ageing Research CARE 75+ study. We have recruited a cohort of older people across the frailty spectrum to investigate frailty transitions and evaluate frailty interventions using a novel cmRCT design [3]. Following recruitment and baseline assessment, participants are followed up at 6, 12, 24 and 48 months. A range of health, social and economic data is collected along with demographic information, and information about social networks. In addition, blood samples are collected for storage in a bio-bank for use in future studies.

Alongside the observational study, the cohort is being used as a recruitment platform for qualitative and quantitative studies. It is intended that once numbers increase in the cohort, it will provide a platform for definitive randomised controlled trials. Initially, intervention studies will be restricted to feasibility testing and pilot randomised controlled trials. A range of possible interventions exist – to date there has been interest from researchers working in the areas of dermatology, diet and nutrition, community infection, social networks and pain.

### Cohort multiple Randomised Control Trials (cmRCT)

The cmRCT method is based on establishing a longitudinal observational cohort from which other (sub) studies can recruit. The original cohort is used as a ‘patient identification site’ (PIC). CmRCTs are best suited to acute clinical conditions and chronic conditions - for which many different types of interventions may be trialled concurrently. They are also beneficial for conditions for which previous trials have struggled with recruitment [4].

Two key features of the cmRCT are ‘active recruitment’ and ‘embedded controls’. Using a model of ‘real world’ or ‘patient centred’ information’ – only those *actively* participating in a sub-study receive patient information. Cohort participants who are not recruited to the intervention arm of a sub-study will have their background observational data used for control purposes (consent for this is sought at the time of original recruitment to the cohort) [4]. In this way, cmRCTs facilitate the inclusion of cognitively impaired participants, and other hard to reach groups, who, as controls, are not required to review new study information.

Although, the inclusion of those with cognitive impairment at the outset is still prescribed by the study design i.e. dependent on the advice of a consultee and not by an adapted trial design, the potential for an individual to be included if they develop cognitive impairment during the lifetime of the study is greater as they will have had an opportunity to make the decision in advance.

In addition, multiple interventions can be trialled concurrently in one cohort. CmRCTs can facilitate meta-analysis using standardised outcome measures across studies - assuming there is an agreed minimum dataset; maximising resources and minimising participant burden.

Historically, clinical trials of interventions for older people are characterised by low recruitment rates for a number of reasons, such as exclusion criteria – in particular cognitive impairment [3], patient preferences, and ethical dilemmas [4]. This has resulted in a limited evidence base for treatments and services for older people. It is therefore expected that using a cmRCT method will improve research in populations of older people and the evidence based outcomes.

Despite the potential of this increasingly popular methodology there is an absence of guidance about how PPIE activity fits with this study design. The Medical Research Council (MRC) guidelines on maximising the value of large cohorts notes ‘stakeholder’ engagement but does not discuss the potential role of the public especially with regard to increasing retention and recruitment [5].^.^ Furthermore, a review of birth cohorts suggest that public engagement (PE) should be ‘strengthened’ [6], but offers little indication of how this could be practicably achieved.

### The need for engagement

The arguments for public involvement in cohort studies are the same as those given for involvement in any health service research. There are moral, political, economic and pragmatic reasons for involving the public and patients in all aspects of research from design to dissemination [7, 8]_._

The national and international importance of involving ‘consumers’ in service design and research has long been recognised [9]. Organisations such as INVOLVE testify to the government’s commitment to public and service user engagement in the research process in the UK [10]. Moreover, most major UK health research funders such as NIHR and MRC advocate public involvement in research or expect valid reasons as to why it has not been included in applications [11, 12].

Most systematic reviews of PPIE conclude that attempts to conceptualise, evaluate and report outcomes are still in their infancy [11, 13]. Nevertheless, there are already many descriptive reports of the benefit of PPIE from individual studies [12, 14, 15].

### Additional considerations for cmRCTs

Over the past 10 years there has been a development of strong values and principles which should underpin PPIE work [16, 17] alongside toolkits which support the practical application of these [18, 19]. Alongside these there is agreement that PPIE cannot be prescriptive and flexibility is needed to ensure the activity is meaningful, appropriate and impactful within any given context. There are numerous examples of good practice from individual studies. However, there is little in the way of guidance about which paradigms might best support specific study types [6, 20]. Whilst briefing notes for researchers present potential benefits and challenges to PPIE in different clinical trial scenarios they do not suggest any specific mechanisms which would work best in these scenarios [21].

We believe there are additional considerations which should influence the structure of PPIE in cohorts used as a platform for multiple studies.

Firstly, it is imperative that there is monitoring and scrutiny of the original aims and objectives of the observational study for which the cohort was developed. For example, when new studies wish to utilise the cohort they may require additional outcome measures or propose duplicating/modifying some existing measures. All changes need to be reviewed carefully and objectively to avoid any unnecessary additional burden on the participants.

Secondly, the recruitment and retention of lay representatives needs to be considered. In longitudinal observational cohort studies, perhaps more than in shorter studies, public representation may be better served by community organisations than by individuals.

Thirdly, as with all cohort populations, older people with frailty are not a homogenous group – there is huge diversity both within and across geographic regions. What is relevant to an older person in Bradford may have little relevance to an older person in Bath. This becomes increasingly important in multi-site studies where recruitment and retention rates can vary enormously [22] and be adversely affected by local context [23, 24] But, whilst there is an acknowledgement that international trials need to consider local context this is normally viewed through the lens of research staff and not patients or the wider public [25]. In addition, reported reasons for differential recruitment across sites include external publicity and conflict with other trials [26], both of which are pertinent in cmRCTs.

Finally, the potential range of topics for sub-studies is vast. Therefore, it is unreasonable to expect the original lay representatives to have either the time or breadth of interest to be involved in the detail of all studies. Sub-studies require their own engagement structures to focus on specific topics, but these need to be linked and co-ordinated in order to reduce duplication and/or contradiction.

### The CARE 75+ cohort involvement model

Within the CLAHRC frailty theme, we were challenged to create a structure to enable meaningful, public involvement within the cmRCT framework. We needed a structure that had the potential to connect us to the whole spectrum of older people with frailty across a range of cohort sub-studies in the future. What we have established is a core reference group of four key individuals with extensive links to other relevant local community structures. Within the Frailty Oversight Group (FOG) we have lay representation from the Bradford Older People’s Partnership [27], the Bradford Self-Care and Prevention Forum and a general practice Patient Participation Group along with a BME advocate from the local authority [28]. This core group undertakes a monitoring and scrutiny role for the original observational study alongside providing oversight for the wider programme of work utilising the cohort within the frailty theme. And, provide signposting to researchers and advocacy for participants.

**Fig. 1:**
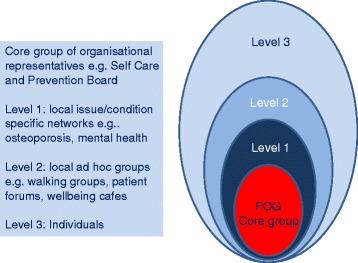
CARE75+ Model of Involvement

At the centre we have recruited from key health and ageing organisations (Fig. 1). We want to keep organisations on board for the duration of the cohort, mindful of the fact that individual representatives may come and go. The representatives from these key organisations have specific expertise of their own. And, are able to network the research team quickly and effectively to other relevant local organisations, groups or individuals depending on the specific needs of the study. In doing so, they are able to extend the PPIE activity for the whole of the Frailty Theme way beyond the scheduled meeting times. They can do this because they have extensive local knowledge and long-standing relationships engendering trust within their communities.

Core organisational members provide stability over time which means the structure is not reliant on individuals. At a secondary level core group members will have links to specialist condition/disease groups provides patient expertise and experience. Finally, at the widest community level, local ad hoc groups and individuals, engaged via the networks provided by the core group members, maintain diversity and can be engaged for one-off consultation activity. However, the various levels are not hierarchical and any of them can be used for consultation, collaboration or co-production interchangeably, as appropriate.

It is important that flexibility is retained and any members of the core group who are interested in sub-studies can be more or less involved as they wish. In encouraging sub-studies to regularly report back to the core group, those members can consider how sub-study decisions might impact the wider programme of work, for example in the methods and timing of recruitment. Sub-study groups may also provide a natural recruitment resource for the core group in future, and those leaving the core group can remain within the structure at a more informal level i.e. level 3 as and when they are interested and able.

Whilst it is important that sub-studies undertake their own, bespoke, PPIE activity, clear branding of the sub-studies will maintain an association with the original cohort. This will improve external publicity – benefiting from PPIE work that has already taken place, and reduce conflict with other trials by fostering a culture of collective responsibility across the studies. In maintaining the brand all PPIE activity will have reach beyond its original aim.

**Fig. 2:**
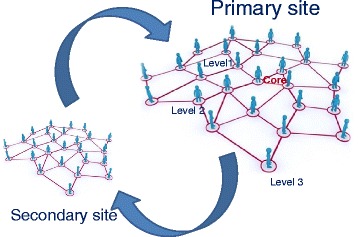
Networked Involvement ‘webs’

Another representation of the FOG structure would be a ‘web’ of interconnectedness (Fig. 2). But, it is not sufficient to have only one ‘web’. Our aim is to replicate the structure in different localities across the study sites to enable us to access local knowledge and needs. Furthermore, we aim to connect each site so that learning can be shared in adequate time across the cohort. This along with central co-ordination will allow wider learning for sub-studies, reduce the burden on the central team and maintain equality across the regions.

The model we have adopted embodies the values and principles of good PPIE in the following ways: The core FOG members meet independent of the Frailty Theme Operational Board. This is to ensure that the focus of the meetings remains with the lay members. The project manager chairs the meetings. This role has been offered to members who so far have declined, but the intention is to offer the role in the future. The PI for the CARE75+ study is always required to attend and all other researchers attend as and when necessary. Researchers advocating all new proposals for use of the cohort are required to attend. Outcomes from the FOG are fed into other arenas such as the overall Frailty Theme Operational Group and study co-ordination meetings.

The core FOG members meet quarterly, although PPIE activity also takes place in the interim, and all discussions are fully documented for reference. Any issues impacting beyond the scope of the group are forwarded to the relevant organisations or groups such as the Integrated Care Board. All actions taken as a result of the FOG are documented in the meeting minutes and followed up. Reasons for in-action are noted. The FOG members have clear terms of reference for the scope of their work. They are also linked to complimentary structures such as the AHSN, Improvement Academy.

Core members are financially reimbursed at a rate of £10 per hour for any time spent engaged in project work e.g. meetings, training, observations and background reading. This is in addition to out of pocket expenses to pay for parking or transport. Lay members have received training in observational methods and briefings in event facilitation and presentations before carrying out the tasks. One of the first requests from lay members was a research language ‘jargon buster’.

Researchers from all sub-studies are required to regularly update the core members of the FOG and make representations if necessary. The fluidity of the FOG model is such that it can accommodate the views of individuals or groups allowing for more diverse representation in the main cohort study and sub-studies.

## Results

### Work undertaken by the Frailty Oversight Group

We have been developing the FOG model for the past 18 months and already there are a number of significant outcomes which evidence the added value of this approach across the whole of the research cycle. In total, over 100 individuals have helped to shape our work.

#### Identifying and prioritising areas of research

During the first study wide ‘Celebration Event’ of the CARE75+ study, 70 participants were asked to prioritise a number of areas of interest to researchers including; pain, loneliness, infection and skin health. The core FOG members were briefed to facilitate this exercise and helped to identify loneliness and pain as important topics to take forward within the cohort. In the following months two grant applications were developed and submitted on those topics.

Skin health was not identified as a priority for the wider cohort participants and within the quarterly meeting the core FOG members were able to drill down to why exactly this was the case, providing the researcher with valuable insight with which to refocus their proposal.

*The FOG had quite an impact on my thinking – particularly their obvious lack of enthusiasm for my work (in the nicest possible way). It’s made me think even more about how I can convince funders that this work is worthwhile – and the need for it to have tangible impact/patient benefit. It’s obvious really – but very powerful when it comes directly from ‘real’ people* (Researcher).

#### Study design

Before fieldwork started within the main cohort, core FOG members were able to source three ‘naive’ dummy participants with whom the researchers could test out the data collection process from start to finish. This enabled the team to go beyond ‘role play’ in their training and help them understand the process from a participant’s point of view. From this experience the researchers were able to demonstrate ‘content/community validity’ [14], were confident in their approach and, able to be realistic and sensitive in scheduling time with participants i.e. allowing more time for questions which could elicit an emotional response. Excerpts from the test feedback illustrate the value of this exercise.

*“I kept talking I did elaborate. I think they’re going to meet this anyway. A lot of people need to talk. They were very good. They had very good listening skills. Offered to stop to have a cup of tea”* (test participant 1).

*“It was very clear. They were extremely polite. They were concerned about my welfare and that everything was alright with me. The opportunity to come back is a good option.”* (test participant 2).

Core members also sourced other individuals with experience of chronic pain to test the proposed data collection tool for the pain study. This was a well-established tool which has been used for a pan-European study of pain. However, within our test-bed of frail older people we found that some of the pain descriptors were not well understood and needed adjustment.

One of the sub-studies used the core members to signpost researchers to groups primarily concerned with supporting older people in the community e.g. British Legion. In this way they were able to develop their own specialist engagement group for the shorter duration of the sub-study.

#### Development of grant proposals

Because of the close links the FOG has with related areas of work within the research unit, one member was seconded to be a co-applicant on a Health Technology Assessment bid addressing extended rehabilitation. As exercise and mobility was one of the top three priorities identified at the Celebration Event this as considered an important programme with which to link.

#### Management

Very early on the lay members identified that researchers should be mindful of not increasing social isolation when withdrawing from assessments. Because of their extensive local networks they were able to suggest organisations that would ensure continuity for the participants and give them an opportunity to start building networks if they wished. These were then added to the existing signposting resource that is made available to the participants.

One of the on-going agenda items for the FOG is how to keep in touch with participants between assessments, which are at 6 and then 12 month intervals. The importance of this contact was highlighted during the observations. To date the group have held one ‘Celebration Event’ and are in the process of planning a second. They have also reviewed and contributed quarterly newsletters.

Another area of interest which the FOG would like to be considered is the issue of ‘drop out’. Core members feel it is important to understand why participants would not wish to continue in the study in order to improve retention rates. This is an area for consideration in the future. However, our data suggests that the study has experienced relatively low attrition rates (6.84 %) compared to community based trials including older people [29].

Local networks and other district wide work with older people enabled FOG members to signpost the research team to a research active GP which resulted in increased Black, Minority Ethnic (BME) participation to 14 %, in line with the local population.

In assuring good governance of the study the FOG members also raised the question of how much support the researchers need/receive when they are faced with participants who may have mental health problems such as depression or dementia or seemingly be very frail and vulnerable. This made the research team consider if the support structures in place for staff were adequate.

#### Undertaking research

Core FOG members have been instrumental in the on-going process evaluation through structured observations of the consent and assessment process. These observations have ensured that the participants’ best interests remain paramount but have also highlighted considerations for the analysis and interpretation of data. For example, the self-report of smoking habits in one case were at odds with the lay members’ observation of the environment. Other points to consider going forward included keeping in touch between assessments, the brief time participants spend reading postal information and appreciating the level of participant nerves at the first encounter.

#### Analysis and interpretation

There have been many discussions about the interpretation of language and concepts at the FOG quarterly meetings. For example, it was noted that the word ‘chronological’ was probably not widely understood and this was removed from a lay summary. In addition, the phrase ‘off legs’ to describe reduced mobility was also not well understood by lay members and was abandoned. However, whilst these individual discussions are useful the real value of the FOG structure lies in sharing them across all of the sub- studies.

Having a multi-layered approach to PPIE has meant that all of the sub- studies have benefitted from shared insight at an early stage. For example, it quickly became apparent from one sub-study that it was not appropriate or meaningful to use the term ‘carer’ for those people providing informal support in the community. This was taken on board by a subsequent study which identified those who provided support as ‘friends, family or neighbours’.

Where issues have been raised in sub-studies researchers have reported these to the core FOG members and they in turn have been able to add another perspective or ratify the original discussion. For example, core members agreed with the sub-study assertion that religious groups should be considered important community support groups. But, the core group did not agree that the sub-study looking at resourcefulness in older people should only focus on non-home owners as a lower income group as there were expenses that could be incurred as a home owner that would put older individuals in a similar financial position to those on low incomes.

#### Dissemination

In addition to their contribution to routine dissemination activities such as the quarterly newsletters and review of the CLAHRC website, FOG members have also co-presented a workshop at one national conference.

However, one of their central roles is in raising awareness of the CARE75+ within the local area through their personal networks. Following an introduction to the Bradford Older People’s forum, researchers from the CARE75+ were able to address a wider audience and elicit views on the shape and scope of the research. This discussion highlighted a potential area of diversity which had been overlooked i.e. inclusion of the long-established Ukranian community in Bradford. In addition, researchers have been invited to present the research at the Bradford Self-Care and Prevention Programme.

## Discussion

Building a model of involvement across time, geographical space and communities of interest has a number of challenges, not least the human resource required to set-up and co-ordinate effective networks at each site. This challenge is not unique to cmRCTs but to any complex studies or large programmes of multi-layered research such as those funded by the CLAHRC or in international trials.

Whilst involvement may have found a place on the agenda in theory, there is still evidence to suggest that researchers will take the path of least resistance and fail to engage patients and the public in a meaningful way, preferring to use what is already in existence in a tokenistic manner [7, 13, 20, 30] rather than build something bespoke. And, although there has been an increase in the amount of resources available to guide researchers in terms of toolkits, values and principles some still struggle to effectively utilise these due to lack of time, resource or confidence in their approach.

We have not found any reference to PPIE models for specific study designs but believe that in the case of cmRCTs and other complex designs a general paradigm is useful in meeting the longitudinal objectives and making the best use of time and resources.

At a time when there is call for the impact of PPIE to be contextualised to provide a more strategic approach [31], practices are not well described in the context of cohort studies. The most extensive review of engagement in cohort studies, which included only birth cohorts describes most engagement activity at the level of information dissemination [6]. Other cohorts offering a facility for ‘trials within cohorts’, such as the South Yorkshire Health Study have only the briefest reference to public engagement on their website [32] and it was confirmed by personal correspondence that this is not an area currently being developed. Other well established ageing cohorts also have no obvious reference to public involvement in their publications or on their websites [33, 34].

Using the FOG model we have been able to involve a significant number of individuals from our target population, i.e. older people aged over 75, across a large spectrum of research activity, in a relatively short space of time. We have both enabled diversity through reaching out via networks into ‘research naive’ communities and encouraged lasting expertise, by skilling up a small core group of collaborators. Because of the inter-connected structure of the FOG, we have been able to strengthen PPIE activity by cross-referencing ideas between the core group, sub-groups and individuals. We have also been able to effectively share the outcomes amongst all researchers working within the Frailty Theme.

Future challenges for the CARE 75+ study oversight are: to establish a network in each of the study sites; to encourage researchers to go beyond the core oversight group, to explore more diverse engagement opportunities and to co-ordinate the work of the local networks to share learning effectively.

## Conclusion

PPIE in cohort studies is imperative and has some unique requirements that are relevant to other complex, multi-layered study designs. The CARE 75+ study proposes one model of PPIE in cmRCTs which provides a structured approach to PPIE in complex and multi-layered study designs, whilst incorporating the values and principles of good practice and maintaining flexibility. Recommendations from the model are shown in Table 1.

**Table 1:** Recommendations for PPIE in CmRCTs

Recommendations	
Choose core group members from established voluntary and community groups which can provide continuity over time	
Ensure all sub-studies protocols are presented to the core group in the initial stages	
Brand sub-studies to ensure they are identified as originating from the cohort	
Use the core group to signpost sub-studies to further engagement opportunities with groups and individuals	
Ensure sub-studies to feedback and share learning quickly	
Establish links with other sites with similar remits	

## Abbreviations

AHSN: Academic Health Science Network
BME: Black Minority Ethnic
CLAHRC: Collaboration for Leadership in Applied Health Research and Care
cmRCT: Cohort multiple Randomised Controlled Trial
FOG: Frailty Oversight Group
MRC: Medical Research Council
PE: Public engagement
PIC: Patient Identification Site
PPIE: Patient and public involvement and engagement
